# Pet Dog Choice in Hong Kong and Mainland China: Exploring Owners’ Motivations, Behaviours, and Perceptions

**DOI:** 10.3390/ani15040486

**Published:** 2025-02-08

**Authors:** Hei Tung Yim, Kate Jade Flay, Omid Nekouei, Paulo Vinicius Steagall, Julia A. Beatty

**Affiliations:** 1 Jockey Club College of Veterinary Medicine and Life Sciences, City University of Hong Kong, Hong Kong SAR, China; 2 Department of Veterinary Clinical Sciences, Jockey Club College of Veterinary Medicine and Life Sciences, City University of Hong Kong, Hong Kong SAR, China; kateflay@cityu.edu.hk (K.J.F.); pmortens@cityu.edu.hk (P.V.S.); 3 Department of Infectious Diseases and Public Health, Jockey Club College of Veterinary Medicine and Life Sciences, City University of Hong Kong, Hong Kong SAR, China; omid.nekouei@cityu.edu.hk; 4 Centre for Animal Health and Welfare, City University of Hong Kong, Hong Kong SAR, China

**Keywords:** canine, human–animal bond, drivers, Asia, rehoming, brachycephalic, trend

## Abstract

Dogs are among the most popular pets worldwide, and ownership is increasing in many regions. Responsible pet ownership positively impacts animal welfare, yet little is known about the drivers of pet choices or their regional variation. To address this knowledge gap, a structured online questionnaire was developed to investigate the motivations, behaviours, perceptions, and demography of owners acquiring dogs. Quality control measures included content validity evaluation by independent experts and back-translation to optimize semantics. Non-probability convenience and purposive sampling was used to recruit respondents through social media and interest groups. In total, 2036 eligible responses were received. Three out of four respondents were female, which is a pattern reported in other regions. Mixed-breed dogs were the most popular, representing around 40% of the total, and non-commercial sources, such as shelters, were the predominant source for new pets (65%). In stark contrast to the UK, USA, and parts of Europe, brachycephalic breeds were the least popular of the purebreds. Poodles were the most frequently owned purebred in both HKSAR and MC. “Companionship for humans” was the most common reason for acquiring a pet dog, and strong agreement with the statement “I consider my dog part of my family” was almost universal (99%). Over half of respondents carried out no research before they acquired their dog (55%), and veterinary professionals were the least frequent resource used by the remainder. Our study reveals commonalities with other regions in drivers for, and perceptions of, pet dogs. Encouragingly from an animal welfare perspective, brachycephalic breeds were the least preferred type. Investigation of the drivers of this observation may be useful to encourage the acquisition of non-brachycephalic dogs in other regions. Overall, our study provides preliminary but valuable insights for developing targeted programs promoting responsible ownership in the region to enhance the well-being of both humans and dogs.

## Simple Summary

Dog ownership can bring a myriad of benefits to both humans and dogs. However, the choice of pet can negatively impact animal welfare if decisions are ill-informed. Factors that influence pet dog choice, which may be region-specific, need to be considered in owner education programs. Little is known about the drivers and demographics of dog ownership in Hong Kong (HKSAR) and mainland China (MC). To address this knowledge gap, an online questionnaire to investigate motivations, behaviours, and perceptions of owners acquiring dogs was designed and validated. In total, 2036 eligible responses were received. Mixed breeds were most popular, representing around 40% of the total, and non-commercial sources, such as shelters, were the predominant source for new pets (65%). In stark contrast to the UK, USA, and parts of Europe, flat-faced (brachycephalic) breeds were the least popular of purebred dogs, whereas Poodles were the most frequently owned purebred in both HKSAR and MC. “Companionship for humans” was the most common reason for obtaining a dog, and strong agreement with the statement “I consider my dog part of my family” was almost universal (99%). Over half of respondents carried out no research before they acquired their dog (55%), and veterinary professionals were the least frequent resource used by the remainder. Our study reveals commonalities with other regions in terms of owners’ drivers for, and perceptions of, pet dogs. Flat-faced breeds were the least preferred type, which is encouraging from an animal welfare perspective since they frequently suffer from severe breathing problems and other diseases. Further investigation of the reasons underlying this observation may be useful to influence pet selection elsewhere. Overall, our study provides preliminary but valuable insights for developing targeted programs promoting responsible ownership in the region to enhance the well-being of both humans and dogs.

## 1. Introduction

Pet ownership is increasing globally, and dogs are a popular pet choice. The role of pet dogs has shifted over time from the fulfilment of working roles to primarily serving as human companions, with dogs increasingly perceived as family members or friends [1,2,3]. The positive benefits of dog ownership for people are well documented and include stress reduction, alleviation of physical and mental health conditions, and companionship [4,5].

Many factors drive decision-making during the acquisition of a pet dog. Prospective owners need to consider factors such as whether to purchase or adopt, the dog’s breed and sex, and their own capacity for ongoing commitment to ownership, such as feeding, exercise, and healthcare [6,7,8,9]. Understanding owners’ acquisition patterns and behaviours during pet selection ultimately impacts dog welfare [6]. The welfare impact for individual animals can be positive, as occurs when an unowned dog is adopted into an environment where its needs are well met. Conversely, the purchase of extreme brachycephalic, or “flat-faced”, breeds fuels a market for dogs that inevitably suffer from poor health [10,11,12,13].

Owners’ motivations have been broadly categorised as either extrinsic or intrinsic when selecting a pet [14]. Extrinsically motivated owners prefer dogs with a specific appearance that they perceive brings personal gains such as social recognition, status, and health benefits [14,15]. Such motivations align with trends in breed popularity, celebrity associations, and owner resemblance [6,16,17]. Extrinsically motivated owners also perceive their dogs as needy or helpless [14]. In contrast, intrinsically motivated owners value their dogs as unique individuals, appreciating their autonomy and perceiving them as friends rather than as dependent children. Intrinsically motivated owners are likely to prioritize animal welfare [14]. Rescuing and rehoming dogs also serve as a primary motivation for some owners, reflecting an ethical stance [18,19].

Physical appearance is a key consideration, particularly when selecting brachycephalic breeds or adopting from shelters [1,6,14,16,20]. Owners of dogs with less extreme conformations exhibit more intrinsic motivation and consider factors such as health of the breeds compared to owners of breeds with more extreme conformations, such as French Bulldogs, Chihuahuas, and Cavalier King Charles Spaniels [16].

The extent to which pet ownership decisions are affected by regional and cultural factors and whether any universal trends exist are unclear. Available estimates suggest that, like other regions, the frequency of dog ownership is increasing in Hong Kong (HKSAR) and mainland China (MC). According to HKSAR census data, over 1.6 million households had at least one dog in 2011, an increase from 1.4 million households in 1998 [21]. Underreporting of owned dogs in HKSAR is possible, since penalties may be imposed for keeping dogs in public housing [22]. Industry reports suggest a similar increasing trend in dog ownership MC, where 46.1% of an estimated 73 million pet owners selected dogs as their preferred species [23]. Despite this growing population of dog owners, little is known about factors that influence their choices. Additionally, there is limited information available on the demographics of dog owners in China such as their gender, age distribution, and related personal experiences. This study aims to address this knowledge gap by investigating the motivations, behaviours, and perceptions of dog owners in HKSAR and MC when choosing their dogs, providing valuable insights for promoting responsible ownership and enhancing the well-being of both humans and dogs.

## 2. Materials and Methods

This research was approved by the Human and Artefacts Ethics Sub-Committee of City University of Hong Kong (HU-STA-00000567). Access to the survey required respondents to provide informed consent and confirm their eligibility.

### 2.1. Survey Development

A questionnaire was designed to investigate the motivations, behaviours, and perceptions of dog owners in HKSAR and MC when acquiring their dogs. The structured questionnaire was arranged in three sections: owner demographic information, information on all dogs in the household, and detailed information about the most recently acquired dog. The questions were refined and adapted from previous studies with modifications reflecting the HKSAR and MC context [1,6,9,24]. The questionnaire was developed in English and then evaluated for relevance, clarity, and appropriateness of questions by a panel of five veterinarians. Each question was assessed independently by each panel member for relevance using a Likert scale from 1 to 4, from irrelevant to highly relevant, and open feedback was also sought [25]. The content validity ratio (CVR) was determined for each question using the following formula:

$$\frac{\mathrm{CVR}=(\mathrm{ne}-N/2)}{\left(N/2\right)}$$

where ne is the number of experts who gave a score of 3 or 4, and N is total number of experts [26,27]. The survey was modified based on comments from the panel, and items with CVR < 1 were reviewed and then deleted or refined. The final survey contained 23 questions, and the average content validity ratio was 0.82. The English version of the final survey is presented in File S1.

Translation and back-translation were performed to ensure accurate semantics in the Traditional and Simplified Chinese and English versions [27]. HKSAR uses predominantly English and Traditional Chinese, whereas Simplified Chinese is in common use in MC. Neither the individuals involved in translation nor those performing back-translation were involved in questionnaire development. The back-translated English surveys were compared with the original and Traditional and Simplified Chinese surveys and were adjusted, where needed, to ensure accuracy and clarity.

### 2.2. Sampling

The questionnaire survey was hosted on an online survey platform, QuestionPro (https://www.questionpro.com), from 15 November 2023 to 19 January 2024. We used non-probability convenience and purposive sampling to recruit respondents, targeting our advertising towards those who were most likely to own dogs. A poster displaying an overview of the study, eligibility requirements, and a QR code linking to the online survey was distributed via social media platforms and chat groups with the assistance of welfare and industry groups and private veterinary clinics. No incentives were provided to participants, and responses were anonymous and voluntary.

To be eligible for inclusion, respondents had to meet the following requirements: 1. be 18-years of age or older, 2. understand written English, Traditional Chinese, or Simplified Chinese, 3. be resident in HKSAR or MC, and 4. currently own at least one dog. Respondents who selected ‘No’ for voluntary consent and eligibility criteria answered ‘Other’ to the question that asked them if they lived in HKSAR or MC or indicated ‘0’ for the number of dog(s) owned were excluded from the study.

### 2.3. Data Analyses

Raw data were imported from QuestionPro into Microsoft 365 Excel. The survey received 332, 217, and 2689 responses in English, Traditional Chinese, and Simplified Chinese, respectively. After applying the eligibility criteria, 2036 respondents were retained, of which 1648 answered all the survey questions. All eligible responses were included in further analyses, regardless of whether all questions were answered. This resulted in a varying number of responses received for different questions. Further, as some questions asked respondents to select “all that apply”, the number of responses for these questions could be greater than the number of respondents where more than one option was selected.

Purebred dogs were classified according to their cephalic type as brachycephalic, mesocephalic, or dolichocephalic. No definitive reference source for this classification exists, and the anatomic parameters used, such as the cephalic index and craniofacial angle, vary between sources [28,29]. Hence, multiple publications and online resources, including Kennel Club Registries of the UK and USA, were consulted and cross-checked to perform this classification [30,31].

The frequency distributions of responses by all demographic variables were presented in tables or figures. The proportion of responses within each category of the variables of interest (e.g., owner and dog demographic variables) were compared using tests of proportions in Stata v18 (StataCorp LLC, College Station, TX, USA).

## 3. Results

### 3.1. Dog Owner Demographics

Most respondents were from MC (1631, 80.1%; HKSAR 405, 19.9%). The majority of respondents were female (76.2%, Table 1), with the proportion of female respondents being equivalent in HKSAR (77%) and MC (76%). Respondents from HKSAR were older than those from MC, with fewer respondents in the 18–25 age group (Table 1). Approximately one-third of respondents in both regions identified as having current or past involvement with the veterinary industry (Table 1). First-time dog owners made up 40.4% of respondents, and this proportion was consistent among HKSAR and MC respondents. The majority of respondents (73.9%) owned one dog, with only 15.3% and 10.8% owning two or three or more dogs, respectively.

### 3.2. Dog Demographics

#### 3.2.1. Breeds

**Figure 1:**
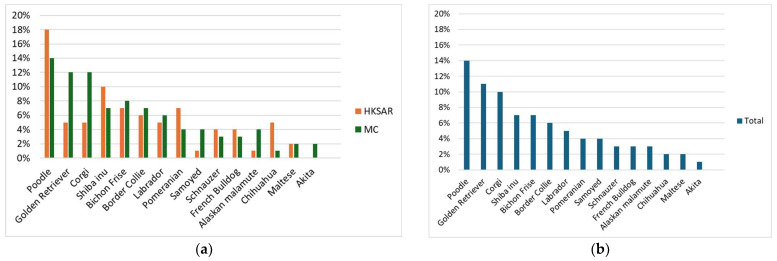
(**a**) Most common purebred dog breeds acquired by respondents in Hong Kong (HKSAR) and mainland China (MC) and (**b**) overall (total). These data are for the respondent’s the most recently acquired dog. The *y*-axis indicates the proportion of respondents that owned each specific breed type.

Respondents were asked about all dogs in the household (Table 2) as well as their most recently acquired dog specifically (Figure 1). Regarding all dogs owned (Table 2), mixed-breed (mongrel) was the most frequent breed type in both locations, with no significant difference in the two proportions (39.2% vs. 41.7%; *p* = 0.299). As shown in Table 2, brachycephalic breeds were the least common in both HKSAR and MC (6.7% overall). However, there was a higher proportion of brachycephalic breeds in HKSAR (12.2%) than in MC (5.0%; *p* < 0.001). A greater proportion of older respondents (>65 years old) owned mixed-breed dogs than younger respondents, and no respondents >65 years old owned brachycephalic breeds. Otherwise, the proportion of respondents that owned brachycephalic breeds increased with increasing age group, from 4.8% to 8.9% (Table 2). There was a slightly higher proportion of brachycephalic breeds owned by females than males (*p* = 0.031). Males were more likely to own mixed-breed dogs than females (*p* < 0.001), while females were more likely to own purebreds (Table 2; All *p* values < 0.05). There was no association between veterinary industry involvement (*p* = 0.593) or first-time dog ownership (*p* = 0.281) and owning a brachycephalic breed.

When the most recently acquired dog was investigated, mixed-breed was again the most popular breed type and brachycephalic breeds the least popular in (Table S1). Interestingly, for recently acquired dogs, mixed-breed was more popular in HKSAR than in MC (166/363 in HKSAR vs. 197/1308 in MC; 45.7% vs. 15.1%; *p* < 0.001). The most frequently owned purebred dogs in HKSAR were Poodle, Shiba Inu, Pomeranian, Chihuahua, Corgi, and Golden Retriever, while in MC, they were Poodle, Golden Retriever, Corgi, Bichon Frise, and Border Collie (Figure 1).

#### 3.2.2. Sex and Neuter Status

The sex and neuter status of dogs owned in HKSAR and MC are presented in Table 3. The proportions of neutered dogs from HKSAR were significantly higher compared to MC in both males (HKSAR = 89.3%, 150/168; MC = 56.2%, 379/674; *p* < 0.001) and females (HKSAR = 92.2%, 153/166; MC = 56.7%, 259/457; *p* < 0.001) (Table 3). Only 6.4% (131/2036) of overall respondents intended to breed their dog, although the proportion of MC respondents that intended to breed their dog (7.4%; 120/1631) was significantly greater than HKSAR respondents (2.7%; 11/405) (*p* < 0.001).

### 3.3. Motivations for Dog Acquisition

Respondents were asked to select all motivations that applied when acquiring their dog, resulting in 3086 responses overall: 685 from HKSAR respondents and 2401 from MC respondents. “Human companionship” was the most frequently selected motivation (1401/3086; 45.4%), followed by “the dog needs a home” (821/3086; 26.6%). There were differences between HKSAR and MC motivations, particularly with regards to “security”; only 2.5% (17/685) of HKSAR responses indicated security as a motivation, compared to 8.1% (194/2401) of MC responses (*p* < 0.001; Table 4).

When considering factors that were influential when owners were making breed choices, there was no clear pattern among respondents, with a number of factors influencing breed choices (Figure S1).

### 3.4. Dog Owner Acquisition Behaviours

#### 3.4.1. Source of Acquisition

Overall, 1686 respondents provided information about the source of the dog that they acquired most recently (Table 5). Most owners (64.5%; 1087/1686) acquired their dogs from non-commercial sources, including “shelter”, “self-bred”, “friends or neighbour”, “stray dog”, and “abandoned by others”. The remaining respondents (35.5%; 599/1686) acquired their dogs from commercial sources, including “pet shop”, “online website”, and “breeder”. There were differences in acquisition patterns between HKSAR and MC respondents, with a greater proportion of MC respondents acquiring their dogs from commercial sources (38.2%; 507/1327) compared with HKSAR respondents (25.6%; 92/359%) (*p* < 0.001). A greater proportion of MC respondents acquired their dog from a pet shop, friend/neighbour, or as stray, while a greater proportion of HKSAR respondents acquired their dog from a shelter (Table 5). A greater proportion of younger respondents (ages 18–40) acquired their dog from commercial sources (38.6%; 526/1361) compared to older respondents (22.3%; 72/323; *p* < 0.01) (Table S2). A greater proportion of female respondents (66.1%) acquired their dog from non-commercial sources compared to male respondents (58.2%) (*p* = 0.008; Table S2). A greater proportion of first-time dog owners acquired their dog from commercial sources compared to those that already owned a dog (39.8% vs. 32.8%; *p* = 0.003).

#### 3.4.2. Resources Consulted Prior to Acquisition

Overall, around half of the respondents (n = 752; 44.8%) sought information about the breed/type of dog prior to acquisition, while the others (n = 926; 55.2%) carried out no research (Table S3). A greater proportion of HKSAR respondents (218/353; 61.8%) carried out research compared to MC respondents (534/1325; 40.3%; Table S3; *p* < 0.001). However, there was no significant relationship between prior research and any of the other owner demographic factors (*p* > 0.05). Among respondents that carried out research, “media” (seeking information through internet searches, websites, books, and magazines) (33.5% of respondents), “experience and advice of others” (seeking information from other dog owners, friends, and neighbours) (32.5% of respondents), and “pet industry” (seeking information from breeders, groomers, or pet shop staff) (21.5% of respondents) were the most frequent sources of information, with “knowledgeable expert”, such as veterinarians and veterinary professionals, being the least frequent source (only 12.5% of respondents).

### 3.5. Perceptions of Dog Owners Regarding Their Dogs

Most respondents (1729/1746; 99.0%) strongly agreed with the statement “I consider my dog part of my family”, with this proportion being high for both HKSAR and MC respondents (Table S4). Similarly, the majority were happy with their choice of most recently owned dog, with 79.1% (1318/1666) selecting “happy” and 16.1% (269/1666) selecting “slightly happy” (Table S5). There was a difference in reported satisfaction between first-time dog owners and those that had prior dog ownership experience (Table S5), with a lower proportion of first-time dog owners indicating “happy” (75.4%; 504/668) compared to those that already owned dogs (81.5%; 813/997) (*p* = 0.003). The majority of owners reported that their dogs were “very healthy” (64.2%) or “healthy” (26.4%) (Table S6). A greater proportion of MC respondents reported their dog as “very healthy” (67.0%) compared to respondents from HKSAR (53.7%) (Table S6; *p* < 0.001). Among owners that reported specific health complaints, the most frequent types were dermatologic and orthopaedic issues followed by obesity and gastrointestinal issues (Table S7). Additionally, 163 owners reported age-related issues with their dogs.

## 4. Discussion

This study generated novel data regarding pet dog ownership in HKSAR and MC and describes factors associated with ultimate pet choice. Consistent with European data [24,32,33], the majority of the survey respondents were female. The age structure of our respondents varied, with HKSAR respondents being older and having an age structure similar to that described in a UK study [24]. The preponderance of younger respondents in MC may reflect that the increase in pet dog ownership is a recent trend [23], whereas dog ownership in HKSAR has been popular for some time [21]. First-time dog owners represented around 40% of all respondents, which is higher than that reported in comparable surveys conducted in the UK [24]. “Companionship for humans” was the most common reason for dog acquisition among our study population, aligning with the results of research reported from New Zealand, Australia, and the UK [6,9,12,34,35].

Overall, mixed-breed dogs were the most commonly owned (around 40% in both locations), contrasting with overseas data, where purebreds are more popular choices [31,36]. Interestingly, brachycephalic breeds were the least popular breed type in both HKSAR and MC, contrasting with the situation in the USA, Australia, and Europe, where ownership of brachycephalic breeds (e.g., Pug, French Bulldog, and English Bulldog) has increased dramatically, such that they now represent a large proportion of the total dog population in these regions [24,31,36,37]. Studies conducted in Australia and the UK hypothesised that increasing brachycephalic ownership may be influenced by a trend towards apartment living, which could be perceived to better support ownership of small-breed dogs [24,36]. In HKSAR and MC, apartment living is the norm, yet brachycephalic breeds were the least popular, suggesting that different drivers may exist in different geographic and cultural communities. However, as we did not ask the respondents about their housing, we were unable to investigate this relationship further. Further investigation of the reasons underlying the relative unpopularity of brachycephalic breeds in HKSAR and MC might inform strategies to reduce acquisition of these breed types in other regions, thereby promoting improved animal welfare. 

Poodles were the most popular purebred here, but we found no clear patterns among the reported factors that influenced breed selection. Female respondents were more likely to own purebred dogs than male respondents. When considering only respondents that owned purebred dogs, the ten most common breeds represented nearly 70% of the purebred dog population for HKSAR and MC. A similar lack of diversity was reported in the UK, where the top ten breeds represented nearly 60% of dogs in the UK [31].

The majority of dog owners acquired their dogs via non-commercial sources, specifically shelters in HKSAR and friends or family in MC. This may contribute to the high proportion of mixed-breed dogs in our study population, as purebred dogs are more likely to be sold by commercial sources such as pet stores, which were the source of only a quarter of the dogs acquired most recently. This contrasts with data from New Zealand, where breeders were the most common source of pet dogs (around 60%) and only 16% of dogs were obtained from animal shelters [34]. Many factors could influence our observation, including logistical considerations, such as the relative availability of dogs from non-commercial sources, or differing motivations for dog ownership between our study population and dog owners in other regions. For instance, owners in the USA display clear preferences for specific breeds or types of dogs, while around one third of potential Australian owners believed that shelter dogs often have behavioural problems, which discouraged them from adopting from shelters or rescue centers [18,38]. In our study population, over a quarter of respondents selected “the dog needed a home” as a motivation for dog acquisition, suggesting an ethical approach to dog ownership. A study conducted in the USA found that women favor adoption or rescue more than men, and women were more likely to own a dog because they had rescued them [18]. The same trend was identified in this study, with a greater proportion of female respondents acquiring their dog from non-commercial sources compared to male respondents.

The proportion of neutered dogs among HKSAR owners was very high (>90%) compared with MC respondents in our study (52.6%), and compared to previous reports of dogs in Italy and Western Europe [32,39]. The high rates of dog acquisition from shelters among HKSAR owners may have contributed to this, as dogs rehomed from shelters are neutered routinely before rehoming. In Italy, the prevention of behavioural problems has been identified as a common reason for neutering, and similar motivations may influence dog owners in HKSAR due to the high population density and apartment living [39]. These same European studies suggests that male dogs are more likely to be neutered than female dogs [32,39], whereas comparable rates of neutering between male and female dogs were identified in HKSAR (89.3% and 92.2%, respectively) and in MC (56.2% and 56.7%, respectively). Interestingly, nearly 16% of owners that responded to the question regarding neutering status were unsure if their dog was neutered, regardless of whether the dog was male or female. This might suggest that neuter status was not considered when the dog was acquired, contrasting an Australia study where neutering status was considered during the acquisition process [40]. Alternatively, the question may have been misunderstood, despite the content validation measures taken here.

Around half (55%) of the respondents carried out no research before they acquired their dog, a higher proportion than has been reported overseas [41,42,43]. Among the respondents that did conduct research, “media”, “pet industry”, and “experience and advice of others” (outside the vet industry, e.g., other dog owners, friends, and neighbours) were the most frequent sources of information, with “knowledgeable expert”, such as veterinarians and veterinary professionals, being the least frequent source. A similar pattern is reported elsewhere, with more than 30% of prospective owners using the internet as their primary information source and only 6.2% of respondents consulting veterinary professionals for advice [43]. Considering the risk of encountering unreliable information online, this highlights a concerning trend. Further investigation is recommended to determine if respondents that rely on internet information or guidance from other dog owners, friends, or family are receiving reliable and appropriate advice [41]. Further, if potential dog owners are encouraged to develop a pre-acquisition relationship with a veterinarian, this may enhance their future care of their dog and lead to better health outcomes.

Almost unanimously, the respondents considered their pet dogs to be family members. Owners who hold this view may be more likely to discover the survey on their social media feeds and/or to respond to it. Nonetheless, our observation is consistent with an international phenomenon, where companion animals are increasingly regarded as family members [34,44,45]. As reported elsewhere [19,35], our respondents were happy with their choice of most recently owned dog, although a difference in reported satisfaction between first-time dog owners and those that had prior dog ownership experience was noted, with a lower proportion of first-time dog owners indicating “happy” compared to those that already owned dog(s). This might suggest that there were aspects of dog ownership that first-time dog owners were unprepared for, in which case, the promotion of preparation (including research) before acquisition may be useful.

This study had limitations in addition to those described previously, including the use of convenience sampling. We chose this sampling method due to its accessibility and ability to provide a high number of responses. Participation was voluntary, and we distributed our questionnaire in online groups primarily consisting of animal support communities and local veterinary clinics, which may inherently pre-select for individuals positively disposed towards animals who are more likely to be engaged in such communities. We were unable to identify the primary forum from which the respondents accessed the survey (i.e., the proportion from different animal support communities or veterinary clinics). We observed differences in the proportion of respondents based on gender. Other similar studies have also reported an overrepresentation of females [12,32,35], which is likely impacted by factors such as an increased likelihood of women to respond to surveys and their interest in animal-related topics [46]. Furthermore, the respondents were asked to answer questions based on their recollection of their dog acquisition process. Their motivations, actions, and dog acquisition processes may have changed over time, and their memory recall may be biased. Other potential sources of bias include non-response bias and social desirability bias [47]. Despite these limitations, our survey collected a relatively high number of responses compared with studies in other regions and provides valuable, novel data in an area that has previously been unexplored in HKSAR and MC.

## 5. Conclusions

The motivations, behaviours, and perceptions of owners uncovered in this study have implications for the welfare of current and future generations of dogs in this region. This research partly addresses the existing knowledge gap in dog ownership in HKSAR and MC, highlighting the need for continued understanding and development of targeted measures to promote responsible dog acquisition and improve the well-being of dogs and support the human–animal bond.

## Figures and Tables

**Table 1:** Frequency distribution of demographic information of 2036 dog owners in Hong Kong (n = 405) and mainland China (n = 1631), with the percentage calculated based on the number of responses in each location (column).

Demographic Variable	Categories	No. of Responses (%)	Hong Kong (%)	Mainland China (%)	*p*-Value ^1^
Age (years)	18–25	702 (34.5)	41 (10.1)	661 (40.5)	<0.001
	26–40	947 (46.5)	172 (42.5)	775 (47.5)	0.068
	41–65	370 (18.2)	182 (44.9)	188 (11.5)	<0.001
	>65	14 (0.7)	9 (2.2)	5 (0.3)	<0.001
	Unanswered	3 (0.1)	1 (0.2)	2 (0.1)	0.559
Gender	Female	1551 (76.2)	312 (77.0)	1239 (76.0)	0.651
	Male	415 (20.4)	86 (21.2)	329 (20.2)	0.635
	Non-binary	14 (0.7)	1 (0.2)	13 (0.8)	0.231
	Unanswered	56 (2.7)	6 (1.5)	50 (3.1)	0.081
Veterinary industry involvement	Yes	588 (28.9)	115 (28.4)	473 (29.0)	0.809
	No	1448 (71.1)	290 (71.6)	1158 (71.0)	0.809
First-time dog owner	Yes	823 (40.4)	153 (37.8)	670 (41.1)	0.225
	No	1211 (59.5)	252 (62.2)	959 (58.8)	0.209
	Unanswered	2 (0.1)	0 (0.0)	2 (0.1)	0.481

^1^ *p*-values are from comparing the proportion of responses within each category between the two locations using tests of proportions.

**Table 2:** Frequency distribution of dog breeds owned by the respondents, classified according to skull type. Respondents were stratified based on overall demographic data, with the percentage calculated based on the number of responses for each category (row).

Demographic Variable	Categories	No. of Responses	Brachycephalic (%)	Mesocephalic (%)	Dolichocephalic (%)	Mixed-Breed ^1^ (%)
Location	Hong Kong	531	65 (12.2)	134 (25.2)	124 (23.4)	208 (39.2)
	Mainland China	1678	84 (5.0)	611 (36.4)	283 (16.9)	700 (41.7)
Age (years)	18–25	735	35 (4.8)	275 (37.4)	115 (15.6)	310 (42.2)
	26–40	1023	74 (7.2)	347 (33.9)	199 (19.5)	403 (39.4)
	41–65	427	38 (8.9)	116 (27.2)	92 (21.5)	181 (42.4)
	>65	18	0 (0.0)	3 (16.7)	1 (5.6)	14 (77.8)
Gender	Female	1228	120 (9.8)	571 (46.5)	329 (26.7)	208 (16.9)
	Male	414	26 (6.3)	149 (36.0)	72 (17.4)	167 (40.3)
	Non-binary	21	0 (0.0)	10 (47.6)	2 (9.5)	9 (42.9)
Veterinary industry involvement	Yes	567	41 (7.2)	193 (34.0)	125 (22.0)	208 (36.7)
	No	1642	108 (6.6)	552 (33.6)	282 (17.2)	700 (42.6)
First-time dog owner	Yes	710	42 (5.9)	262 (36.9)	133 (18.7)	273 (38.5)
	No	1497	107 (7.1)	482 (32.2)	274 (18.3)	634 (42.4)

^1^ As the skull shape of mixed-breed dogs is unknown, they are reported separately.

**Table 3:** Sex and neuter status of 2036 dogs owned in Hong Kong (HKSAR) and mainland China (MC). Each respondent provided detailed information about their most recently acquired dog.

Sex	Neuter Status	Overall No. of Responses	No. of Responses HKSAR	No. of Responses MC
Male	Entire	313	18	295
(n = 999)	Neutered	529	150	379
	Unsure	157	17	140
Female	Entire	211	13	198
(n = 742)	Neutered	412	153	259
	Unsure	119	20	99
Unanswered (n = 295)			34	261

**Table 4:** The five most frequent motivations for dog acquisition, stratified based on owner demographic information: location, age, gender, veterinary industry involvement, and previous dog ownership, with the percentage calculated based on the number of responses for each category (row).

Demographic Variable	Categories	No. of Responses	Human Companion (%)	Needs a Home (%)	Pet Companion (%)	Security (%)	Children (%)
Location	Hong Kong	685	284 (41.5)	212 (30.9)	83 (12.1)	17 (2.5)	40 (5.8)
	Mainland China	2401	1117 (46.5)	609 (25.4)	178 (7.4)	194 (8.1)	164 (6.8)
Age (years)	18–25	1095	516 (47.1)	268 (24.5)	90 (8.2)	99 (9.0)	63 (5.8)
	26–40	1385	658 (47.5)	367 (26.5)	114 (8.2)	88 (6.4)	71 (5.1)
	41–65	579	219 (37.8)	178 (30.7)	55 (9.5)	21 (3.6)	67 (11.6)
	>65	23	7 (30.4)	7 (30.4)	2 (8.7)	3 (13.0)	2 (8.7)
Gender	Female	2339	1066 (45.6)	647 (27.7)	193 (8.3)	154 (6.6)	144 (6.2)
	Male	647	285 (44.0)	149 (23.0)	60 (9.3)	47 (7.3)	56 (8.7)
	Non-binary	18	9 (50.0)	3 (16.7)	2 (11.1)	3 (16.7)	1 (5.6)
Veterinary industry involvement	Yes	946	381 (40.3)	270 (28.5)	82 (8.7)	73 (7.7)	73 (7.7)
	No	2140	1020 (47.7)	551 (25.7)	179 (8.4)	138 (6.4)	131 (6.1)
First-time dog owner	Yes	1179	584 (49.5)	278 (23.6)	81 (6.9)	82 (7.0)	75 (6.4)
	No	1906	816 (42.8)	543 (28.5)	180 (9.4)	129 (6.8)	129 (6.8)

**Table 5:** Sources of acquisition of pet dogs from Hong Kong (n = 359) and China (n = 1327), with the percentage calculated based on the number of responses in each location (column).

Source	Categories	Overall No. of Responses (%)	Hong Kong (%)	Mainland China (%)	*p*-Value ^1^
Commercial	Pet shop	432 (25.6)	56 (15.6)	376 (28.3)	<0.001
	Website	62 (3.7)	9 (2.5)	53 (4.0)	0.184
	Breeder	105 (6.2)	27 (7.5)	78 (5.9)	0.253
Non-commercial	Shelter	367 (21.8)	198 (55.2)	169 (12.7)	<0.001
	Self-bred	27 (1.6)	3 (0.8)	24 (1.8)	0.193
	Friend or neighbour	589 (34.9)	49 (13.6)	540 (40.7)	<0.001
	Stray	78 (4.6)	9 (2.5)	69 (5.2)	0.031
	Abandoned by others	26 (1.5)	8 (2.2)	18 (1.4)	0.234

^1^ *p*-values are from comparing the proportion of responses within each category between the two locations using tests of proportions.

## Data Availability

The original contributions presented in this study are included in the article/Supplementary Material. Further inquiries can be directed to the corresponding author.

## Supplementary Materials

The following supporting information can be downloaded at: https://www.mdpi.com/article/10.3390/ani15040486/s1, File S1: Survey used to explore motivations, behaviours and perceptions of owners acquiring a dog in Hong Kong or mainland China. Figure S1. Frequency of factors (% of responses) that influenced dog owners when making breed selection during the dog acquisition process, stratified based on owner demographic information. Table S1: Frequency distribution of dog breeds most recently acquired by the respondents, classified according to skull type; Table S2. Source of acquisition of pet dogs for 1686 dog owners, stratified based on owner demographic information, with the percentage calculated based on the number of responses for each category (row).; Table S3. Frequency of research prior to dog acquisition for 1678 dog owners, stratified based on owner demographic information, with the percentage calculated based on the number of responses for each category; Table S4. Agreement with the statement “I consider my dog part of my family” among dog owners, stratified based on owner demographic information, with the percentage calculated based on the number of responses for each category; Table S5. Owner reported satisfaction with their choice of most recently acquired dog, stratified based on owner demographic information, with the percentage calculated based on the number of responses for each category; Table S6. Owner reported health of their most recently acquired dog, stratified based on owner demographic information, with the percentage calculated based on the number of responses for each category; Table S7. Type and frequency of health complaints from dog owners (n = 329) who scored their dog as “2—Healthy” to “5—Very unhealthy”, ordered from most to least frequently reported.

## References

[1] Packer R.M.A., Brand C.L., Belshaw Z., Pegram C.L., Stevens K.B., O’Neill D.G. (2021). Pandemic Puppies: Characterising Motivations and Behaviours of UK Owners Who Purchased Puppies during the 2020 COVID-19 Pandemic. Animals.

[2] Greenebaum J. (2004). It’s a Dog’s Life: Elevating Status from Pet to “Fur Baby” at Yappy Hour. Soc. Anim..

[3] Blouin D.D. (2013). Are Dogs Children, Companions, or Just Animals? Understanding Variations in People’s Orientations toward Animals. Anthrozoös.

[4] Barker S.B., Wolen A.R. (2008). The benefits of human–companion animal interaction: A review. J. Vet. Med. Educ..

[5] Powell L., Chia D., McGreevy P., Podberscek A.L., Edwards K.M., Neilly B., Guastella A.J., Lee V., Stamatakis E. (2018). Expectations for dog ownership: Perceived physical, mental and psychosocial health consequences among prospective adopters. PLoS ONE.

[6] Holland K.E. (2019). Acquiring a Pet Dog: A Review of Factors Affecting the Decision-Making of Prospective Dog Owners. Animals.

[7] Aylesworth A., Chapman K., Dobscha S. (1999). Animal companions and marketing: Dogs are more than just a cell in the bcg matrix!. Adv. Consum. Res..

[8] Tesfom G., Birch N.J. (2013). Does definition of self predict adopter dog breed choice?. Int. Rev. Public Nonprofit Mark..

[9] Holland K.E., Mead R., Casey R.A., Upjohn M.M., Christley R.M. (2022). Why Do People Want Dogs? A Mixed-Methods Study of Motivations for Dog Acquisition in the United Kingdom. Front. Vet. Sci..

[10] Fawcett A., Barrs V., Awad M., Child G., Brunel L., Mooney E., Martinez-Taboada F., McDonald B., McGreevy P. (2019). Consequences and Management of Canine Brachycephaly in Veterinary Practice: Perspectives from Australian Veterinarians and Veterinary Specialists. Animals.

[11] Mitze S., Barrs V.R., Beatty J.A., Hobi S., Bęczkowski P.M. (2022). Brachycephalic obstructive airway syndrome: Much more than a surgical problem. Vet. Q..

[12] Packer R.M.A., O’Neill D.G., Fletcher F., Farnworth M.J. (2019). Great expectations, inconvenient truths, and the paradoxes of the dog-owner relationship for owners of brachycephalic dogs. PLoS ONE.

[13] O’Neill D.G., Pegram C., Crocker P., Brodbelt D.C., Church D.B., Packer R.M.A. (2020). Unravelling the health status of brachycephalic dogs in the UK using multivariable analysis. Sci. Rep..

[14] Beverland M.B., Farrelly F., Lim E.A.C. (2008). Exploring the dark side of pet ownership: Status- and control-based pet consumption. J. Bus. Res..

[15] Wood L., Martin K., Christian H., Nathan A., Lauritsen C., Houghton S., Kawachi I., McCune S. (2015). The Pet Factor—Companion Animals as a Conduit for Getting to Know People, Friendship Formation and Social Support. PLoS ONE.

[16] Sandøe P., Kondrup S.V., Bennett P.C., Forkman B., Meyer I., Proschowsky H.F., Serpell J.A., Lund T.B. (2017). Why do people buy dogs with potential welfare problems related to extreme conformation and inherited disease? A representative study of Danish owners of four small dog breeds. PLoS ONE.

[17] Zhang X., He Y., Yang S., Wang D. (2024). Human Preferences for Dogs and Cats in China: The Current Situation and Influencing Factors of Watching Online Videos and Pet Ownership. Animals.

[18] Bir C., Widmar N.J.O., Croney C.C. (2017). Stated Preferences for Dog Characteristics and Sources of Acquisition. Animals.

[19] Blackman S.A., Wilson B.J., Reed A.R., McGreevy P.D. (2019). Reported Acquisition Practices of Australian Dog Owners. Animals.

[20] Weiss E., Miller K., Mohan-Gibbons H., Vela C. (2012). Why Did You Choose This Pet?: Adopters and Pet Selection Preferences in Five Animal Shelters in the United States. Animals.

[21] Census and Statistics Department (2019). Thematic Household Survey Report No. 66.. Government of the Hong Kong Special Administrative Region: Website of the Census and Statistics Department. www.censtatd.gov.hk.

[22] Tilley H.B., Ho S.P., Woodhouse F., Whitfort A. (2025). Population Estimates and the Effect of Trap-Neuter Return Program on the Free-Roaming Dog Population in Hong Kong SAR. J. Appl. Anim. Welf. Sci..

[23] Xu H., Yang J. (2019). Understanding Dog Owners’ Decision Making on Dog-Related Consumption In China. Master’s Thesis.

[24] Packer R.M.A., Murphy D., Farnworth M.J. (2017). Purchasing popular purebreds: Investigating the influence of breed-type on the pre-purchase motivations and behaviour of dog owners. Anim. Welf..

[25] Joshi A., Kale S., Chandel S., Pal D.K. (2015). Likert scale: Explored and explained. Br. J. Appl. Sci. Technol..

[26] Almanasreh E., Moles R., Chen T.F. (2019). Evaluation of methods used for estimating content validity. Res. Soc. Adm. Pharm..

[27] Luna S.P.L., Trindade P.H.E., Monteiro B.P., Crosignani N., della Rocca G., Ruel H.L.M., Yamashita K., Kronen P., Tseng C.T., Teixeira L. (2022). Multilingual validation of the short form of the Unesp-Botucatu Feline Pain Scale (UFEPS-SF). PeerJ.

[28] Ekenstedt K.J., Crosse K.R., Risselada M. (2020). Canine Brachycephaly: Anatomy, Pathology, Genetics and Welfare. J. Comp. Pathol..

[29] Carreira L., Ferreira A. (2015). Reference values for dog sagittal and transverse cephalic indices in different skull types and their importance. J. Anim. Vet. Adv..

[30] McGreevy P.D., Georgevsky D., Carrasco J., Valenzuela M., Duffy D.L., Serpell J.A. (2013). Dog Behavior Co-Varies with Height, Bodyweight and Skull Shape. PLoS ONE.

[31] O’Neill D.G., McMillan K.M., Church D.B., Brodbelt D.C. (2023). Dog breeds and conformations in the UK in 2019: VetCompass canine demography and some consequent welfare implications. PLoS ONE.

[32] Kubinyi E., Turcsán B., Miklósi Á. (2009). Dog and owner demographic characteristics and dog personality trait associations. Behav. Process..

[33] Steinert K., Kuhne F., Kramer M., Hackbarth H. (2019). People’s perception of brachycephalic breeds and breed-related welfare problems in Germany. J. Vet. Behav..

[34] Gates M.C., Walker J., Zito S., Dale A. (2019). Cross-sectional survey of pet ownership, veterinary service utilisation, and pet-related expenditures in New Zealand. N. Z. Vet. J..

[35] Dinwoodie I.R., Zottola V., Kubitz K., Dodman N.H. (2022). Selection Factors Influencing Eventual Owner Satisfaction about Pet Dog Adoption. Animals.

[36] Teng K.T., McGreevy P.D., Toribio J.-A.L.M.L., Dhand N.K. (2016). Trends in popularity of some morphological traits of purebred dogs in Australia. Canine Genet. Epidemiol..

[37] American Kennel Club Most Popular Dog Breeds. https://www.akc.org/expert-advice/news/most-popular-dog-breeds-by-city-2023/.

[38] Mornement K., Coleman G., Toukhsati S., Bennett P. (2012). What Do Current and Potential Australian Dog Owners Believe about Shelter Practices and Shelter Dogs?. Anthrozoös.

[39] Carvelli A., Scaramozzino P., Iacoponi F., Condoleo R., Della Marta U. (2020). Size, demography, ownership profiles, and identification rate of the owned dog population in central Italy. PLoS ONE.

[40] King T., Marston L.C., Bennett P.C. (2009). Describing the ideal Australian companion dog. Appl. Anim. Behav. Sci..

[41] Kuhl C.A., Lea R.G., Quarmby C., Dean R. (2022). Scoping review to assess online information available to new dog owners. Vet. Rec..

[42] Mead R., Holland K.E., Casey R.A., Upjohn M.M., Christley R.M. (2023). “Do your homework as your heart takes over when you go looking”: Factors associated with pre-acquisition information-seeking among prospective UK dog owners. Animals.

[43] Philpotts I., Blackwell E.J., Dillon J., Tipton E., Rooney N.J. (2024). What Do We Know about Dog Owners? Exploring Associations between Pre-Purchase Behaviours, Knowledge and Understanding, Ownership Practices, and Dog Welfare. Animals.

[44] Noske B. (1997). Speciesism, anthropocentrism, and non-Western cultures. Anthrozoös.

[45] Laurent-Simpson A. (2017). Considering Alternate Sources of Role Identity: Childless Parents and Their Animal “Kids”. Sociol. Forum.

[46] Sax L.J., Gilmartin S.K., Bryant A.N. (2003). Assessing Response Rates and Nonresponse Bias in Web and Paper Surveys. Res. High. Educ..

[47] Krumpal I. (2013). Determinants of social desirability bias in sensitive surveys: A literature review. Qual Quant.

