# Supplementary material for: Pet Dog Choice in Hong Kong and Mainland China: Exploring Owners’ Motivations, Behaviours, and Perceptions
Source: Animals (Basel). 2025 Feb 8;15(4):486. doi: 10.3390/ani15040486 (PMC11851363; doi:10.3390/ani15040486)
Supplement: Supplementary file 1 [file animals-15-00486-s001.zip › animals-3455432-supplementary.pdf]

Pet Dog Choice in Hong Kong and Mainland China: Exploring Owners’ Motivations, Behaviours, and Perceptions

Supplementary materials: Pet dog ownership in Hong Kong and Mainland China: exploring owners’ choices, motivations, behaviours, and perceptions

File S1: Survey used to explore motivations, behaviours and perceptions of owners acquiring a dog in Hong Kong or mainland China.

Survey Questions

There are no RIGHT or WRONG answers to any question in this questionnaire.

Section 1

About yourself

1. **Age: Which age group do you belong to?**
- 18-25
- 26-40
- 41-65
- $\geq$ 65

2. **What is your gender identity?**
- Male
- Female
- Non-binary
- Prefer not to say

3. **I live in:**
- HKSAR
- Mainland China
- Other

4. **Have you ever contributed to the veterinary industry? (e.g., veterinarian, student, nurse, supportive staff, volunteer)**
- Yes
- No

5. **Are you a first-time dog owner?**
- Yes
- No

Section 2

About the pets in your home

6. **What type(s) of dog(s) do you own? (**Tick all that apply)

- ☐ Mixed breed (mongrel)
- ☐ Specific breed
- ☐ Not sure

7. **If you own a specific breed/type of dog(s), please select ALL the breed(s) of your dog(s) from the following list.**

If your breed is not listed, you can add it below.

- ☐ Akita
- ☐ Alaskan malamute
- ☐ American Bulldogs
- ☐ Beagle
- ☐ Bichon Frise
- ☐ Border Collie
- ☐ Boxers
- ☐ Boston Terrier
- ☐ Cavalier King Charles Spaniel
- ☐ Chihuahua
- ☐ Chow Chow
- ☐ Cocker Spaniel
- ☐ Corgi
- ☐ Dachshund
- ☐ Dalmatian
- ☐ Doberman
- ☐ English Springer Spaniel
- ☐ French Bulldog
- ☐ German Shepherd
- ☐ Golden Retriever
- ☐ Great Dane
- ☐ Greyhound
- ☐ Husky
- ☐ Kelpie
- ☐ Labrador
- ☐ Lhasa Apso
- ☐ Malinois
- ☐ Maltese
- ☐ Miniature Schnauzer
- ☐ Pekingese
- ☐ Pug
- ☐ Pointer
- ☐ Pomeranian
- ☐ Poodle (Miniature)
- ☐ Poodle (Standard)
- ☐ Poodle (Toy)
- ☐ Russell Terrier
- ☐ Samoyed
- ☐ Shar Pei
- ☐ Shiba Inu
- ☐ Standard Schnauzer
- ☐ Staffordshire Bull Terrier
- ☐ Tibetan Mastiff
- ☐ West Highland White Terrier
- ☐ Yorkshire Terrier

- ☐ Other

Section 3
About ONE of your dogs
*If you own more than one dog, please answer ALL further questions for the dog you got *most recently**

About your most recently acquired dog.

8. **What was the age when you acquired him/her?**

9. **How old is this dog now?**

10. **What is the type of your MOST RECENTLY acquired dog?**

- Mixed breed (mongrel)
- Specific breed
- Not sure

11. **If your most recently acquired dog is a specific breed/type, please select the breed from the drop-down menu.**

If your breed is not listed, you can add it below.

- Akita
- Alaskan malamute
- American Bulldogs
- Beagle
- Bichon Frise
- Border Collie
- Boxers
- Boston Terrier
- Cavalier King Charles Spaniel
- Chihuahua
- Chow Chow
- Cocker Spaniel
- Corgi
- Dachshund
- Dalmatian
- Doberman
- English Springer Spaniel
- French Bulldog
- German Shepherd
- Golden Retriever
- Great Dane
- Greyhound
- Husky
- Kelpie
- Labrador
- Lhasa Apso
- Malinois
- Maltese
- Miniature Schnauzer
- Pekingese

- Pug
- Pointer
- Pomeranian
- Poodle (Miniature)
- Poodle (Standard)
- Poodle (Toy)
- Russell Terrier
- Samoyed
- Shar Pei
- Shiba Inu
- Standard Schnauzer
- Staffordshire Bull Terrier
- Tibetan Mastiff
- West Highland White Terrier
- Yorkshire Terrier
- Other

12. What is the sex of your most recent dog?
- Male
- Female

13. Has this dog been neutered?
- Yes
- No
- Plan to neuter
- Not sure

14. Will you, or have you, breed/bred from this dog?
- Yes
- No
- Not sure

15. Where did you acquire this dog?
- Pet shop
- Website
- Visited a breeder
- Dog shelter/rehoming organisation (e.g. SPCA)
- I bred this dog myself
- Friend/neighbor
- Other (please specify)

16. **What was your motivation for acquiring this dog?** *Tick all that apply*.

☐ Companionship for humans
☐ Companionship for another pet

- ☐ Security
- ☐ For the children
- ☐ To get fit
- ☐ The dog needed a home
- ☐ Easy to maintain
- ☐ Other (freeform)

17. **Please indicate to what extent you agree with the following statement:**

"I consider my dog part of my family."

Agree	Partly agree	Neutral	Partly disagree	Disagree

18. **How much did the following factors influence your choice to get this breed/type of dog in particular?**

	Not at all	Some influence	Strong influence
Cost of the dog			
Positive experience with this breed/ type of dog before			
This breed/type is generally healthy			
This breed/type is very popular			
This breed/type is a good companion			
Appearance of the breed			
This breed/type needs lots of exercise			
This breed/type needs less exercise			
Someone I know has this breed/type			
I saw this breed/type on social media			
This breed/type is associated with high social status			
I want a small dog			
I want a medium/large sized dog			

If there are other reasons for acquiring this dog, you can tell use here:

(freeform)

19. **Did you do any research prior having a dog?**

- Yes
- No

20. **If yes, what sources did you use to research about your dog's breed/type and how much did you use these resources?**

	Not at all	1 (Used very little)	2	3	4	5 (Used very much)

Online research/websites						
Talking to friends/family						
Talking to a breeder						
Talking to other dog owners						
Talking to a vet/vet staff						
Books/magazines						
Pet shop groomers/staffs						
Other						

If you use *"Other"* resources, please specify:

21. **How happy are you with your choice of dog?** (scale 1-5, 1 is happy, 5 unhappy)

	1 (Happy)	2 (Slightly happy)	3 (Neutral)	4 (Slightly unhappy)	5 (Unhappy)
Rate					

22. **How healthy is your dog?** (scale 1-5, 1 is very healthy, 5 very unhealthy)

	1 (Very healthy)	2 (Healthy)	3 (Neutral)	4 (Unhealthy)	5 (Very unhealthy)
Rate					

23. **If there is anything else you would like to tell us about your dog, please do so now** (freeform)

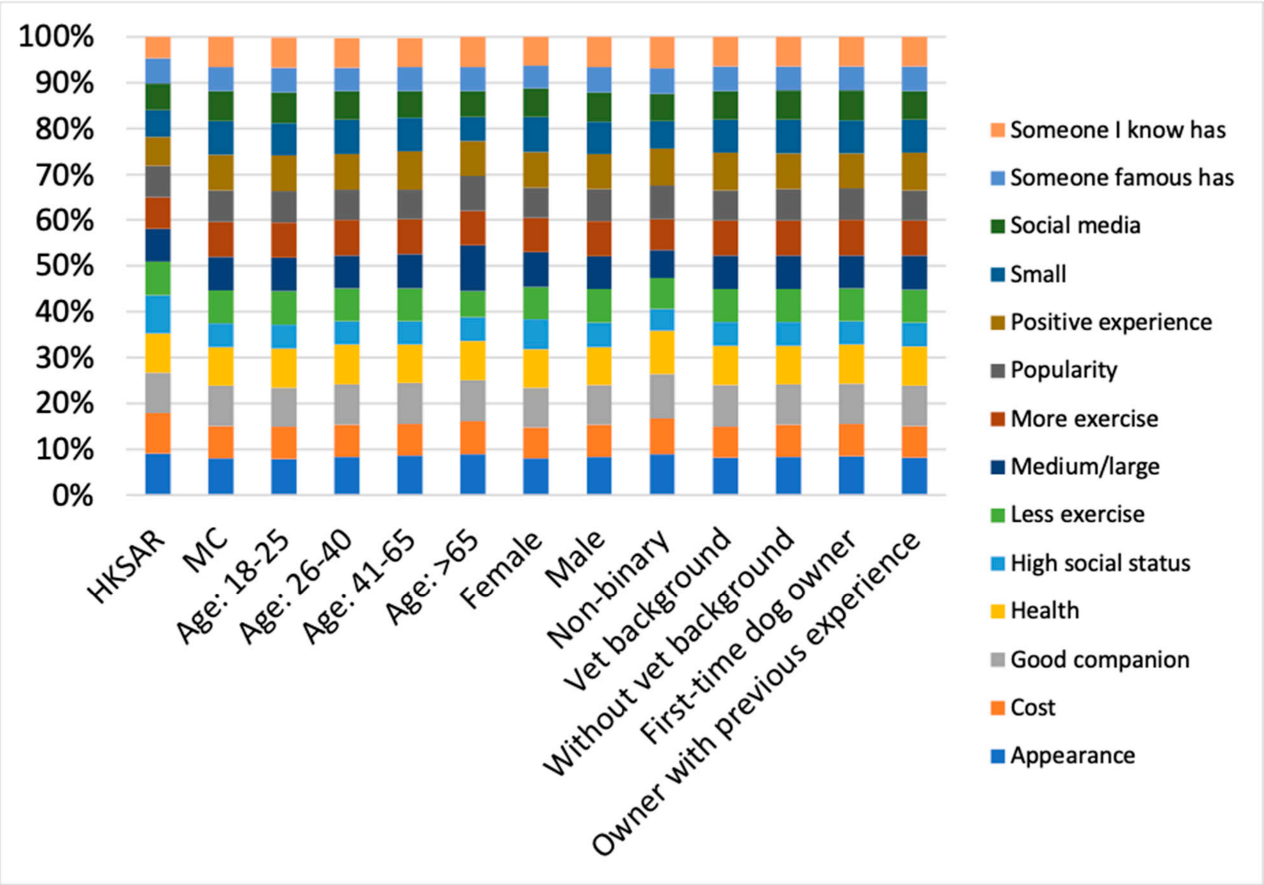

Figure S1. Frequency of factors (% of responses) that influenced dog owners when making breed selection during the dog acquisition process, stratified based on owner demographic information. Factors that respondents could select: "Someone I know has this breed/type"; "Someone famous has this breed/type"; I saw this breed/type on social media"; "I want a small dog"; "Positive experience with this breed/type of dog before"; "This breed/type is very popular"; "This breed/type needs lots of exercise"; "I want a medium/large sized dog"; "This breed/type needs less exercise"; "This breed/type is associated with high social status"; "This breed/type is generally healthy"; "This breed/type is a good companion"; "Cost of the dog"; "Appearance of the breed"

Table S1. Frequency distribution of dog breeds most recently acquired by the respondents, classified according to skull type. Respondents are stratified based on overall demographic data, with the percentage calculated based on the number of responses for each category (row).

Demographic variable	Categories	No. of responses	Brachycephalic (%)	Mesocephalic (%)	Dolichocephalic (%)	Mixed-breed (%)
Location	Hong Kong	363	39 (10.7)	72 (19.8)	75 (20.7)	166 (45.7)
	Mainland China	1,308	55 (4.2)	530 (40.5)	213 (16.3)	197 (54.3)
Age (years)	18 – 25	506	20 (4.0)	224 (44.3)	72 (14.2)	190 (37.5)
	26 – 40	755	50 (6.6)	295 (39.1)	147 (19.5)	263 (34.8)
	41 – 65	298	23 (7.7)	77 (25.8)	69 (23.2)	129 (43.3)
	> 65	12	0 (0.0)	2 (16.7)	0 (0.0)	10 (83.3)
Gender	Female	1,228	73 (5.9)	458 (37.3)	233 (19.0)	464 (37.8)
	Male	294	18 (6.1)	124 (42.2)	50 (17.0)	102 (40.3)
	Non-binary	8	0 (0.0)	2 (25.0)	2 (25.0)	4 (50.0)
Veterinary industry involvement	Yes	455	33 (7.3)	171 (37.6)	78 (17.1)	173 (38.0)
	No	1,130	67 (5.9)	432 (38.2)	211 (18.7)	420 (37.2)
First-time dog owner	Yes	631	39 (6.2)	265 (42.0)	115 (18.2)	212 (33.6)
	No	1,090	67 (6.1)	432 (39.6)	211 (19.4)	380 (34.9)

Table S2. Source of acquisition of pet dogs for 1,686 dog owners, stratified based on owner demographic information, with the percentage calculated based on the number of responses for each category (row).

**Demographic variable**	**Categories**	**Acquisition source**	
		Commercial[1] (%)	Non-commercial (%)
Location	Hong Kong (n = 359)	92 (25.6)	267 (74.4)
	Mainland China (n = 1327)	507 (38.2)	820 (61.8)
Age (years)	18-25 (n = 552)	219 (39.7)	333 (60.3)
	26-40 (n = 809)	307 (37.9)	502 (62.1)
	41-65 (n = 311)	69 (22.2)	242 (77.8)
	>65 (n = 12)	3 (25.0)	9 (75.0)
Gender	Female (n = 1308)	443 (33.9)	865 (66.1)
	Male (n = 323)	135 (41.8)	188 (58.2)
	Non-binary (n = 10)	2 (20.0)	8 (80.0)
Veterinary industry involvement	Yes (n = 482)	155 (32.2)	327 (67.8)
	No (n = 1204)	444 (36.9)	760 (63.1)
First-time dog owner	Yes (n = 681)	271 (39.8)	410 (60.2)
	No (n = 1004)	328 (32.7)	676 (67.3)

[1] Commercial sources included "pet shop", "online website" and "breeder" while non-commercial sources were "shelter", "self-bred", "friends or neighbour", "stray dog" and "abandoned by others".

Table S3. Frequency of research prior to dog acquisition for 1,678 dog owners, stratified based on owner demographic information, with the percentage calculated based on the number of responses for each category (row).

**Demographic variable**	**Categories**	**Pre-acquisition research**	
		Yes (%)[1]	No (%)
Location	Hong Kong (n = 353)	218 (61.8)	135 (38.2)
	Mainland China (n = 1325)	534 (40.3)	791 (59.7)
Age (years)	18-25 (n = 553)	255 (46.1)	298 (53.9)
	26-40 (n = 798)	354 (44.4)	444 (55.6)
	41-65 (n = 314)	140 (44.6)	174 (55.4)
	>65 (n = 11)	1 (9.1)	10 (90.9)
Gender	Female (n = 1300)	583 (44.8)	717 (55.2)
	Male (n = 321)	153 (47.7)	168 (52.3)
	Non-binary (n = 10)	0 (0.0)	10 (100.0)
Vet industry involvement	Yes (n = 474)	235 (49.6)	239 (50.4)
	No (n = 1204)	607 (50.4)	597 (49.6)
First time dog owner	Yes (n = 677)	313 (46.2)	439 (53.8)
	No (n = 1000)	439 (43.9)	561 (56.1)

[1]Sources of research included "media" (seeking information through internet searches, websites, books and magazines), "experience and advice of others" (seeking information from other dog owners, friends and neighbours), "pet industry" (seeking information from breeders, groomers or pet shop staff) and "knowledgeable expert" (seeking information from veterinarians and veterinary professionals)

Table S4. Agreement with the statement "I consider my dog part of my family" among dog owners, stratified based on owner demographic information, with the percentage calculated based on the number of responses for each category (row).

**Demographic variable**	**Categories**	**Agreement**[1]				
		1	2	3	4	5
		n (%)	n (%)	n (%)	n (%)	n (%)
Location	Hong Kong (n = 367)	357 (97.3)	5 (1.4)	3 (0.8)	2 (0.5)	0 (0.0)
	Mainland China (n = 1379)	1313 (95.2)	54 (3.9)	8 (0.6)	3 (0.2)	1 (0.1)
Age	18-25 (n = 575)	555 (96.5)	16 (2.8)	3 (0.5)	1 (0.2)	0 (0.0)
	26-40 (n = 827)	791 (95.6)	31 (3.7)	3 (0.4)	1 (0.1)	1 (0.1)
	41-65 (n = 329)	309 (93.9)	12 (3.6)	5 (1.5)	3 (0.9)	0 (0.0)
	>65 (n = 13)	13 (100.0)	0 (0.0)	0 (0.0)	0 (0.0)	0 (0.0)
Gender	Female (n = 1356)	1314 (96.9)	33 (2.4)	8 (0.6)	1 (0.1)	0 (0.0)
	Male (n = 331)	299 (90.3)	26 (7.9)	2 (0.6)	4 (0.1)	0 (0.0)
	Non-binary (n = 10)	10 (100.0)	0 (0.0)	0 (0.0)	0 (0.0)	0 (0.0)
Vet industry involvement	Yes (n = 500)	470 (94.0)	21 (4.2)	4 (0.8)	4 (0.8)	1 (0.2)
	No (n = 1246)	1200 (96.3)	38 (3.0)	7 (0.6)	1 (0.1)	0 (0.0)
First time dog owner	Yes (n = 701)	673 (96.0)	24 (3.4)	3 (0.4)	1 (0.1)	0 (0.0)
	No (n = 1044)	996 (95.4)	35 (3.4)	8 (0.8)	4 (0.4)	1 (0.1)

[1] Indicates respondent agreement with the statement "I consider my dog part of my family", where: 1 = Agree; 2 = Partly agree; 3 = Neutral; 4 = Partly disagree; 5 = Disagree

Table S5. Owner reported satisfaction with their choice of most recently acquired dog, stratified based on owner demographic information, with the percentage calculated based on the number of responses for each category (row).

Demographic variable	Categories	Satisfaction[1]				
		1	2	3	4	5
		n (%)	n (%)	n (%)	n (%)	n (%)
Location	Hong Kong (n = 347)	293 (84.4)	42 (12.1)	8 (2.3)	3 (0.9)	1 (0.3)
	Mainland China (n = 1319)	1025 (77.7)	227 (17.2)	58 (4.4)	5 (0.4)	4 (0.3)
Age	18-25 (n = 555)	435 (78.4)	89 (16.0)	26 (4.7)	3 (0.5)	2 (0.4)
	26-40 (n = 785)	613 (78.1)	138 (17.6)	31 (3.9)	2 (0.3)	1 (0.1)
	41-65 (n = 313)	259 (82.7)	40 (12.8)	9 (2.9)	3 (1.0)	2 (0.6)
	>65 (n = 11)	10 (90.9)	1 (9.1)	0 (0.0)	0 (0.0)	0 (0.0)
Gender	Female (n = 1293)	1033 (79.9)	197 (15.2)	53 (4.1)	6 (0.5)	4 (0.3)
	Male (n = 317)	237 (74.8)	66 (20.8)	12 (3.8)	2 (0.6)	0 (0.0)
	Non-binary (n = 10)	10 (100.0)	0 (0.0)	0 (0.0)	0 (0.0)	0 (0.0)
Vet industry involvement	Yes (n = 473)	382 (80.8)	64 (13.5)	24 (5.1)	3 (0.6)	0 (0.0)
	No (n = 1193)	936 (78.5)	205 (17.2)	42 (3.5)	5 (0.4)	5 (0.4)
First-time dog owner	Yes (n = 668)	504 (75.4)	130 (19.5)	26 (3.9)	6 (0.9)	2 (0.3)
	No (n = 997)	813 (81.5)	139 (13.9)	(4.0)	2 (0.2)	3 (0.3)

[1] Indicates respondent agreement with the statement "How happy are you with your choice of dog?" Where: 1 = Happy; 2 = Slightly happy; 3 = Neutral; 4 = Slight unhappy; 5 = Unhappy

Table S6. Owner reported health of their most recently acquired dog, stratified based on owner demographic information, with the percentage calculated based on the number of responses for each category (row).

**Demographic variable**	**Categories**	**Dog health status**[1]				
		1	2	3	4	5
		n (%)	n (%)	n (%)	n (%)	n (%)
Location	HKSAR (n = 341)	183 (53.7)	104 (30.5)	33 (9.7)	13 (3.8)	8 (2.3)
	China (n = 1311)	878 (67.0)	332 (25.3)	77 (5.9)	15 (1.1)	9 (0.7)
Age	18-25 (n = 549)	370 (67.4)	136 (24.8)	35 (6.4)	7 (1.3	1 (0.2)
	26-40 (n = 779)	499 (64.1)	218 (28.0)	44 (5.6)	13 (1.7)	5 (0.6)
	41-65 (n = 313)	186 (59.4)	80 (25.6)	29 (9.3)	8 (2.6)	10 (3.2)
	>65 (n = 9)	5 (55.6)	1 (11.1)	2 (22.2)	0 (0.0)	1 (11.1)
Gender	Female (n = 1282)	813 (63.4)	339 (26.4)	93 (7.3)	25 (2.0)	12 (0.9)
	Male (n = 314)	210 (66.9)	85 (27.1)	13 (4.1)	2 (0.6)	4 (1.3)
	Non-binary (n = 10)	7 (70.0)	2 (20.0)	0 (0.0)	0 (0.0)	1 (10.0)
Vet industry involvement	Yes (n = 473)	288 (60.9)	136 (28.8)	36 (7.6)	10 (2.1)	3 (0.6)
	No (1179)	773 (65.6)	300 (25.4)	74 (6.3)	18 (1.5)	14 (1.2)
First-time dog owner	Yes (n = 660)	429 (65.0)	177 (26.8)	37 (5.6)	12 (1.8)	5 (0.8)
	No (n = 991)	632 (63.8)	259 (26.1)	73 (7.4)	16 (1.6)	11 (1.1)

[1] Indicates respondent agreement with the statement "How healthy is your dog?"
Where: 1 = Very healthy; 2 = Healthy; 3 = Neutral; 4 = Unhealthy; 5 = Very unhealthy

Table S7. Type and frequency of health complaints from dog owners (n=329) who scored their dog as "2 – Healthy" to "5 – Very unhealthy", ordered from most to least frequently reported.

Health Complaint	Frequency	Percentage
Dermatologic	70	14.2
Orthopedic	67	13.6
Obesity	40	8.1
Gastrointestinal issues	38	7.7
Dental issues	28	5.7
Cardiologic	21	4.3
Ophthalmologic	15	3.0
Neoplastic	9	1.8
Pancreatic	9	1.8
Respiratory issues	9	1.8
Neurologic	8	1.6
Urinary	8	1.6
Metabolic	5	1.0
Reproductive	2	0.4
